# Unintended pregnancies among HIV-positive women in sub-Saharan Africa: a scoping review protocol

**DOI:** 10.1186/s13643-023-02168-7

**Published:** 2023-01-21

**Authors:** Racheal Tomilola Oguntade, Elizabeth Bolanle Ojewole, Modupe Olufunmilayo Ogunrombi

**Affiliations:** 1 Discipline of Pharmaceutical Sciences, College of Health Sciences, University of Kwa-Zulu Natal, Durban, South Africa; 2 Department of Clinical Pharmacology, School of Medicine, Sefako Makgatho Health Sciences University, Ga-Rankuwa, South Africa

**Keywords:** Prevalence, Incidence, Unintended pregnancies, Contraceptives, HIV-positive women, Sub-Saharan Africa

## Abstract

**Background:**

Unintended pregnancies pose a severe threat to the well-being of HIV-positive women and their unborn children. Factors contributing to the high incidence of unintended pregnancies include contraceptive failure, low uptake of contraceptives, and misuse of contraceptives. Despite various contraceptive options, an increased incidence of unintended pregnancies is rampant among HIV-positive women in the region of sub-Saharan Africa. This study seeks to present evidence of unintended pregnancies among women living with HIV in sub-Saharan Africa, including those using contraceptives.

**Method:**

This study entails a scoping review to survey and interrogate the literature to provide evidence for the incidence of unintended pregnancies among HIV-positive women in sub-Saharan Africa. A proposed framework by Arksey and O’Malley will guide this scoping review. Peer-reviewed articles which address the research questions will constitute the main search. Electronic databases such as EBSCOhost, Cochrane Library, World of Science, World Health Organization (WHO) library databases, Science Direct, Google Scholar PubMed, and gray literature search will be involved. Reference list from studies included will also be searched. The investigation of articles will be done employing keywords from the studies included. The inclusion and exclusion criteria will guide two separate reviewers with the screening of abstracts and full papers. To summarize the findings from this review, thematic content analysis will be done using NVivo version 11.

**Discussion:**

We expect that this review will add to the current body of knowledge on the incidence of unintended pregnancies among HIV-positive women, identify gaps for further future research, and show evidence that may contribute to strengthening the health system’s regulations, guidelines, and policies that may help prevent unintended pregnancies among HIV-positive women.

**Systematic review registration:**

10.17605/OSF.IO/EY3R5

## Background

Unintended pregnancy is a major global public health challenge that negatively impacts women, their children, and society [1]. Unintended pregnancy is that which is mistimed or not wanted at all [2, 3]. There was an estimated 44% unintended pregnancies in 2010–2014 globally. Fifty-nine percent of these unintended pregnancies resulted in abortion in developed regions, as did 55% of unintended pregnancies in developing regions [4].

Over 70% of the approximately 38 million people living with HIV worldwide are in SSA, and most HIV infections occur in this region [5, 6]. Women are disproportionately affected by HIV as about 7000 young females between ages 15 and 24 were newly infected every week in 2017, accounting for three in five new infections globally [7]. Additionally, women are constantly faced with the threat of maternal morbidity and mortality [8]. In 2017, an estimated maternal mortality ratio (MMR) in low-income countries was 462 per 100 000 live births compared to 11 per 100,000 live births in high-income countries [9, 10].

Unintended pregnancy is a common phenomenon among HIV-positive women, an issue of concern [11]. In 2018, 90% of the 1.3 million pregnant women with HIV globally hailed from sub-Saharan Africa (SSA) [12, 13]. Most HIV-positive women stated their most recent pregnancies were unintended, and some have indicated contraceptive use at the time of conception [14–18]. Also, HIV-positive women have more risks posed to their health than their HIV-negative counterparts. They are more likely to die from pregnancy-related complications in addition to the dangers of infecting their unborn children [19]. More so, most HIV infections in young children are acquired via mother-to-child transmission (MTCT) [20–22]. The issue is more prominent in sub-Saharan African countries because over 80% of children infected with HIV are situated in this region [23]. Unfortunately, unintended pregnancies are accompanied by myriads of adverse outcomes such as maternal depression, low birth weight, intimate partner violence, preterm birth, tobacco use during pregnancy, and infant mortality [24].

HIV-positive women are prone to a greater risk of morbidity and mortality during pregnancy and motherhood [19]. They are also more likely to suffer from a severe illness from sexually transmitted infections [25].

Although the initiation of antiretroviral therapy (ART) has helped improve the quality of life of HIV-positive women [26, 27], it also has associated challenges. Studies have shown the adverse impacts of antiretroviral drugs on pregnant women and their neonates; preterm birth and low birth weight have been reported [19, 28].

Furthermore, several studies have reported drug interactions between certain ARVs and hormonal contraceptives (HCs) [29–31].

This scoping review mainly aims to map the evidence of unintended pregnancies occurring among HIV-positive women in SSA. We anticipate this scoping review will present the current prevalence, incidence, and risk factors of unintended pregnancies among HIV-positive women. It is also expected that this scoping review will expose literature gaps for valuable research that may influence policy decisions and means to reduce the impact of unintended pregnancies among HIV-positive women in SSA.

## Methodology

A scoping review maps the literature on the available topic to recognize critical concepts, theories, sources of evidence, and gaps in literature [32, 33]. It helps to know the nature, range, and extent of research available on a subject, summarize, and circulate the findings across a body of research evidence [34]. Therefore, a scoping review synthesizes the literature to provide an overview of what is available in the research topic area as evidence [35, 36].

The methodological framework proposed by Arksey and O’Malley (2005) will be adopted for this proposed review. Therefore, the following five stages will be followed in this scoping review: (i) recognizing the research question, (ii) recognizing relevant studies, (iii) selection of eligible studies, (iv) charting the data, and (v) collating, summarizing, and reporting the results. This current scoping review intends to chart all research activities in this area; therefore, a quality appraisal will not be carried out.

### Recognizing the research question

The main research question this review seeks to provide an answer to is “what is the evidence that HIV-positive women experience unintended pregnancies, including those that may be on contraceptives?”

The research sub-questions are as follows:

1. What is the evidence of the incidence of unintended pregnancies among HIV-positive women of reproductive age?
2. What is the evidence of the incidence of unintended pregnancies among HIV-positive women of reproductive age on contraception?
3. What are the contributing factors to unintended pregnancies among HIV-positive women of reproductive age?

### Recognizing relevant studies

Published and unpublished (gray) literature will be explored on unintended pregnancies among HIV-positive women using electronic databases including Cochrane Library, World of Science, PubMed, and WorldCat. Studies reported from the year 2000 until December 2022 will be included. The Medical Subject Headings (MeSH) terms or keywords will include “HIV positive women,” “unintended pregnancies,” “contraception,” “sub-Saharan Africa (SSA),” “low- and middle-income countries,” “unplanned birth,” “unwanted pregnancy,” “unplanned pregnancy,” “unintended births,” and “unwanted births.” The appropriateness of keywords and databases will be ascertained by piloting the search strategy. To ensure no vital information is left out, keywords may be refined to include different groups of HIV-positive women, such as those using antiretrovirals (ARVs). A hand search will also be conducted of the references cited in the included studies and search on websites including World Health Organization (WHO) to identify other potentially relevant literature. Potentially relevant gray literature will be identified through specific searches of dissertations/theses (ProQuest Dissertations & Theses Global) and conference abstracts. The PEO framework (Table 1) will guide title and abstract screening. Further eligibility criteria will ensure that the content of the included studies is relevant to the research question.

**Table 1:** A PEO framework for eligible studies

P–population	HIV-positive women of reproductive age (15–49 years) in SSA
E–exposure	Unintended pregnancy
O–outcome	Prevalence Incidence Risk factors of unintended pregnancies

### Selection of eligible studies

#### Inclusion criteria

Studies must meet the following criteria to be included:

- HIV-positive women of reproductive age who experienced unintended pregnancies
- Published from the year 2000 to December 2022
- Qualitative and quantitative studies
- Sub-Saharan African countries (SSA)
- Articles written in English language

### Exclusion criteria

Studies must have the following characteristics to be excluded:

- Studies without HIV-positive women of reproductive age
- Studies where the researcher could not get the full-text article

**Fig. 1:**
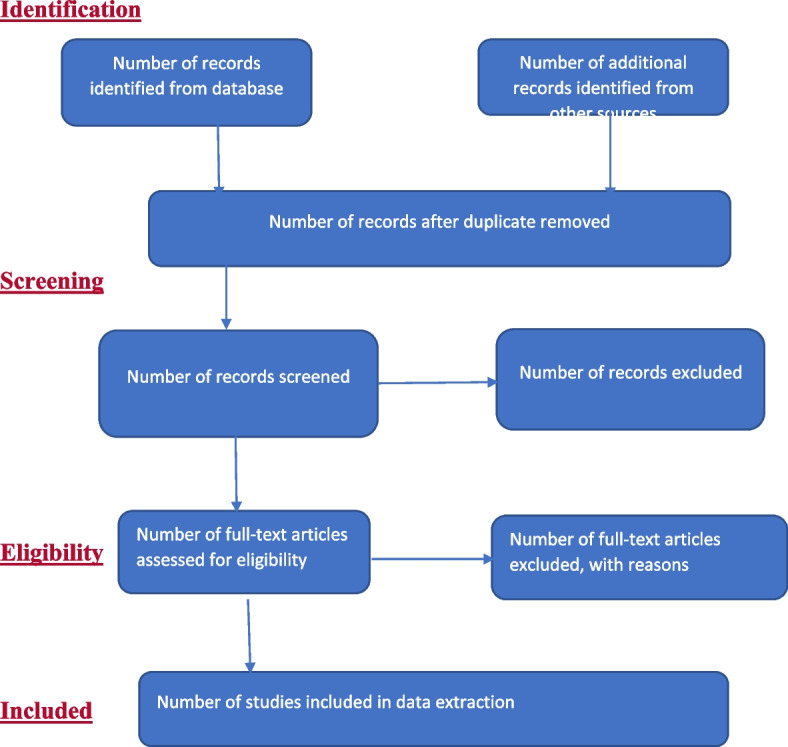
Study selection flow diagram as described by Arksey and O’Malley [32]

All eligible articles will be imported into Mendeley Desktop software following a comprehensive search using the keywords mentioned earlier. Duplicate reports will be identified and removed. Two separate reviewers will screen the title and abstract of the eligible articles to ascertain if they fit the review. The full-text screening will also be done (see Fig. 1). There will be the involvement of a third reviewer should there be any discrepancies at the stage of full-text screening. The full text of selected articles will be gotten by making all possible efforts to either search the Internet, intreating with the UKZN librarian, or reach the author if need be. Search record details such as search date, database, number of studies identified, keywords, and number of studies eligible will be appropriately documented. The guideline to report screening results will be adopted from the recommendations in the Preferred Reporting Items for Systematic Reviews and Meta-Analyses Extension for Scoping Reviews (PRISMA-ScR) [37]. Mapping will be done using the PRISMA-P chart [38]. Tables 2 and 3 will be used to present the results of the titles searched from various databases.

**Table 2:** Records of the database searched

Date of search	Search engine used	Keyword searched	Number of articles found	Number of eligible articles

**Table 3:** Pilot search in PubMed database

Date of search	Search engine used	Keyword searched	Number of publications retrieved
17/12/22 PubMed		("pregnancy, unplanned"[MeSH Terms] OR ("pregnancy"[All Fields] AND "unplanned"[All Fields]) OR "unplanned pregnancy"[All Fields] OR ("unintended"[All Fields] AND "pregnancy"[All Fields]) OR "unintended pregnancy"[All Fields]) AND ("hiv seropositivity"[MeSH Terms] OR ("hiv"[All Fields] AND "seropositivity"[All Fields]) OR "hiv seropositivity"[All Fields] OR ("hiv"[All Fields] AND "positive"[All Fields]) OR "hiv positive"[All Fields]) AND ("women"[MeSH Terms] OR "women"[All Fields]) AND ("epidemiology"[Subheading] OR "epidemiology"[All Fields] OR "prevalence"[All Fields] OR "prevalence"[MeSH Terms]) AND ("risk factors"[MeSH Terms] OR ("risk"[All Fields] AND "factors"[All Fields]) OR "risk factors"[All Fields]) AND ("epidemiology"[Subheading] OR "epidemiology"[All Fields] OR "incidence"[All Fields] OR "incidence"[MeSH Terms]) AND ("africa south of the sahara"[MeSH Terms] OR ("africa"[All Fields] AND "south"[All Fields] AND "sahara"[All Fields]) OR "africa south of the sahara"[All Fields] OR ("sub"[All Fields] AND "saharan"[All Fields] AND "africa"[All Fields]) OR "sub saharan africa"[All Fields])	2094

### Charting the data

Relevant information will be extracted from each of the studies included utilizing a data charting form, generated electronically using Google forms. The data extracted will consist of the following (Table 4):

**Table 4:** Data extraction form

Author and date of publication Study title Study aim/objective Type of study design Study setting Study population Sampling method Data collection methods Data analysis method Significant findings Conclusion	

### Collating, summarizing, and reporting the results

A narrative approach will present the findings from the studies that meet the inclusion criteria via thematic content analysis. NVivo version 11.0 software will be employed for the extraction themes that are relevant to the study. Collation, summary, and reporting of themes will focus on the outcomes of prevalence, incidence, and risk factors of unintended pregnancy among HIV-positive women. Also, emerging themes will be reported. These results will be described in accordance with the overall purpose of the research and help identify knowledge gaps.

## Discussion

This proposed review is intended to map evidence for the occurrence of unintended pregnancies among HIV-positive women of reproductive age in SSA. It will also help identify risk factors for unintended pregnancies among HIV-positive women and present the burden of unintended pregnancies among HIV-positive women.

Most HIV-positive women have indicated that their pregnancies were unintended [14, 18]. Some reported using contraception at the time of conception [14, 16, 17]. Understanding contraception among HIV-positive women will help make inferences on the menace of unintended pregnancies and suggest practical recommendations for prevention in the nearest future. It will also be a pointer to support further research on contraceptive use among women living with HIV.

## Conclusion

This review will show the evidence of unintended pregnancies among HIV-positive women in SSA. The findings of this scoping review will provide helpful information to undertake further research that will help generate the evidence that may be needed to make informed decisions regarding contraceptive use among HIV-positive women in SSA.

## Data Availability

All data generated and analyzed in this research will constitute the scoping review article.

## Abbreviations

HIV: Human immunodeficiency virus
SSA: Sub-Saharan Africa
WHO: World Health Organization
MMR: Maternal Mortality Ratio
MTCT: Mother-to-child transmission
MeSH: Medical Subject Headings
ARVs: Antiretrovirals
PEO: Population, Exposure, Outcome
PRISMA-ScR: Preferred Reporting Items for Systematic Reviews and Meta-Analyses Extension for Scoping Reviews
PRISMA-P: Preferred Reporting Items for Systematic Review and Meta-analysis Protocol
