# Correction: Unintended pregnancies among HIV‑positive women in sub‑Saharan Africa: a scoping review protocol

**DOI:** 10.1186/s13643-023-02336-9

**Published:** 2023-09-04

**Authors:** Racheal Tomilola Oguntade, Elizabeth Bolanle Ojewole, Modupe Olufunmilayo Ogunrombi

**Affiliations:** 1 Discipline of Pharmaceutical Sciences, College of Health Sciences, University of Kwa-Zulu Natal, Durban, South Africa; 2 Department of Clinical Pharmacology, School of Medicine, Sefako Makgatho Health Sciences University, Ga‑Rankuwa, South Africa

**Correction:** **Systematic Reviews 12, 12 (2023)**

10.1186/s13643-023-02168-7

Following publication of the original article [1], the author reported that author names were incorrectly presented. Given names and family names were switched and are presented correctly in this correction article.

The incorrect author names are:

Oguntade Racheal Tomilola, Ojewole Elizabeth Bolanle and Ogunrombi Modupe Olufunmilayo

The correct author names are:

Racheal Tomilola Oguntade, Elizabeth Bolanle Ojewole and Modupe Olufunmilayo Ogunrombi

The original article [1] has been corrected.
